# Policy Gaps and Opportunities in Bio-Based Plastics: Implications for Sustainable Food Packaging

**DOI:** 10.3390/foods14111955

**Published:** 2025-05-30

**Authors:** Walter Leal Filho, Jelena Barbir, Madhavi Venkatesan, Amanda Lange Salvia, Andrea Dobri, Neda Bošković, João Henrique Paulino Pires Eustachio, Ian Ingram, Maria Alzira Pimenta Dinis

**Affiliations:** 1 Research and Transfer Centre Sustainability & Climate Change Management (FTZ-NK), Hamburg University of Applied Sciences, 21033 Hamburg, Germanyandrea.dobri@haw-hamburg.de (A.D.);; 2 Department of Economics, Northeastern University, 360 Huntington Avenue, Boston, MA 02115, USA; 3 Graduate Program in Civil and Environmental Engineering, University of Passo Fundo, BR 285, São José 99052-900, PF, Brazil; 4 Institute for Interdisciplinary and Multidisciplinary Studies, University of Montenegro, Cetinjski put b.b, Podgorica 81000, Montenegro; nedaboskovic93@gmail.com; 5 Department of Natural Sciences, Manchester Metropolitan University, John Dalton Building, Chester Street, Manchester M1 5DG, UK; 6 Fernando Pessoa Research, Innovation and Development Institute (FP-I3ID), University Fernando Pessoa (UFP), Praça 9 de Abril 349, 4249-004 Porto, Portugal; madinis@ufp.edu.pt; 7 Marine and Environmental Sciences Centre (MARE), University of Coimbra, Edifício do Patronato, Rua da Matemática, 49, 3004-517 Coimbra, Portugal

**Keywords:** bio-based plastics, food packaging, plastic pollution, sustainable materials, toxicity, policy, circular economy

## Abstract

The increasing use of bio-based and/or biodegradable plastics reflects a global push towards more sustainable materials. In the context of food packaging, where plastic waste and contamination risks are acute, these materials offer promising alternatives. However, the transition is complex, requiring coordinated regulatory interventions and lifecycle assessments (LCA) to avoid unintended environmental and health consequences. This paper outlines the pressing need for policies that guide the development and deployment of bio-based plastics in food-related applications. It provides a policy-oriented synthesis focused on Europe and discusses recent concerns such as toxicity, end-of-life impacts, and food safety. The study draws from the literature review and regulatory analysis to suggest policy mechanisms that can accelerate safe, circular solutions in food packaging. Methodologically, this communication uses qualitative synthesis of scientific and regulatory data to assess gaps and align innovations with sustainability targets.

## 1. Introduction: Plastic Pollution and Food Packaging Contexts

This article uses a qualitative synthesis of the recent scientific literature, European Union (EU) policy documents, and regulatory frameworks, with the goal to critically evaluate the role of bio-based plastics in food packaging and propose strategic policy directions for sustainable development. This discussion distinguishes between the broad ecological impacts of plastic pollution and the specific challenges posed by plastics in food packaging contexts, where issues of toxicity, contamination, and consumer exposure are especially acute. The article contributes to the literature by identifying critical policy gaps and actionable opportunities in bio-based plastics for food packaging, offering evidence-based recommendations to align regulatory frameworks with sustainability goals.

During the last 50 years, the use of synthetic, petroleum-derived plastic has significantly increased [1]. The lightweightness, flexibility, and durability of these plastics have in large part facilitated their ubiquitous, albeit [1,2,3,4] not sustainable, presence [5].

Ninety-nine percent of plastics are dependent on fossil fuels, with a significant share, estimated at 50 percent of plastic products, designed for single-use [6]; 36 percent [7,8] of these are used in the food industry (e.g., food wrap, food containers). The use of these products unfortunately appears to have only considered the myopic use value of convenience without addressing the overall present and intertemporal impacts of plastic. Quite simply, plastics are not, in practice, easily disposed of.

Inasmuch as the majority of plastics are not recycled or reused, at disposal these products are a direct ecosystem threat in the form of litter, with the attribution of causation of death to animals on land and water, inclusive of birds [3,5,9]. Records of wildlife ingesting plastic go as far back as 1957 [10]. The United Nations estimates that plastic is responsible for the deaths of more than a million seabirds and over 100,000 marine mammals every year [11]. Further, many animals die or are harmed due to entanglement in plastic debris or ghost fishing gear. Table 1 summarizes some of the major environmental consequences associated with plastic pollution. Though exact percentage attribution has not been assessed, there is sufficient evidence to link these adverse environmental impacts to plastics used for food products [12].

Further, given the composition of plastic and its synthetic properties, it does not degrade in an environmentally aligned manner; estimates are that petroleum-based plastics take a minimum of 450 years [13] to photodegrade naturally in the environment, which means they stay in the environment forever—they will not biodegrade. Increasingly, research on the impact of plastic use has surfaced the emerging threat to human health as well. The channels of human ingestion include consumption of animals, fruits, and vegetables, as well as air and water [14]. This includes evidence of bioaccumulation in the human body and brain and evidence of plastic crossing the human placenta [15,16,17]. Further and related to life on the planet, petroleum-based plastics contribute to climate change [18,19,20]. Plastic production is expected to only increase global greenhouse gas emissions, with an attribution given to a four percent increase in annual production of “25–31% of the remaining global carbon budget for limiting global warming to 1.5 °C” [21].

Landfilled plastic will likely never degrade, while incinerated plastics create ambient toxins as dioxins [22,23]. According to the World Health Organization, dioxins are a known carcinogen and are responsible for cancer in animals and likely in humans [24]. Dioxins may also cause other reproductive or developmental effects. Further, as incineration relies on a continuous stream of waste, it is arguably not a deterrent to waste creation; it potentially justifies it [25]. Recycling is expensive because plastic comes in many different varieties, requiring each type of plastic to be recycled by a different process. Further, recycling is a downstream, consumer-oriented activity; since more waste occurs in the production process, recycling is insufficient to mitigate overall plastic waste [26]. As of 2015, of the 8.3 billion metric tons of plastic that have been produced, 6.3 billion metric tons have become plastic waste. Of that, only 9% has been recycled, 12% was incinerated, and 79% was accumulated in landfills or the natural environment [27]. In addition to the 5 trillion pieces of plastic floating in the world’s five ocean gyres, multiples of that amount are estimated to have been deposited on the ocean floor [27,28].

Beyond the challenges associated with the persistence and accumulation of plastics in the environment, a further risk is posed by the additives that are often incorporated into plastics to enhance their properties. Some of the problems associated with the additives used in plastics are summarized in Table 2. Arguably, the use of plastics in food production, storage, and consumption. introduces unique concerns both related to significance in use and the limited understanding of impacts to the human body.

**Table 1:** Environmental concerns linked to plastic pollution, including risks to aquatic life and food chain contamination.

Problem	Impacts	Reference
Pollution of rivers and seas	Degradation of plastic releases and contaminants	[29,30,31]
Damage to fauna	Injuries to birds and aquatic animals entangled into plastic pieces and nets	[32,33]
Disposal in beaches	Negative consequences to landscapes	[34,35]
Ingestion by fish	Lands in the food chain, reaching humans	[36,37]
Consumption by birds	Starvation, suffocation, drowning	[38,39]

Note: Recent scientific studies have identified microbial strains capable of degrading certain conventional plastics such as polyethylene terephthalate or polyethylene under laboratory conditions, although such processes are not yet scalable or widely adopted [4]. Source: authors.

**Table 2:** Toxicity risks of commonly used plastic additives, with potential relevance for food-contact materials.

Additive Type	Commonly Used Substances	Reference	EC/List No	Hazard Classification and Labelling by to ECHA in CLP
1. Functional				
Plasticizers (10–70) *	1,2-Benzene-dicarboxylic acid, di-C6-8-branched alkyl esters, C7-rich (DIHP)	[40]	276-158-1	** According to the CLP (ATP01 for DIHP and ATP17 for boric acid) approved by the European Union, these substances may damage fertility and may damage the unborn child. Substances predicted as likely to meet criteria for category 1A or 1B carcinogenicity, mutagenicity, or reproductive toxicity, or with dispersive or diffuse use(s) where predicted likely to meet any classification criterion for health or environmental hazards, or where there is a nanoform soluble in biological and environmental media.
Flame retardants (3–25) *	Boric acid	[41]	234-343-4	
Stabilizer (0.5–3) *	Lead and lead compounds	[42]	231-100-4	** According to the ECHA in REACH registrations, this substance may damage fertility or the unborn child, may cause harm to breast-fed children and causes damage to organs through prolonged or repeated exposure. Is very toxic to aquatic life with long-lasting effects, may cause cancer. Some data submitters indicate they consider this substance as carcinogenic.
2. Colorants (0.01–10) *	Cadmium and cadmium compounds	[43]	231-152-8	** According to the ECHA in REACH registrations, this substance is fatal if inhaled, is very toxic to aquatic life, may cause cancer, causes damage to organs through prolonged or repeated exposure, is suspected of causing genetic defects, is suspected of damaging fertility or the unborn child and catches fire spontaneously if exposed to air. These substances are carcinogenic, suspected to be mutagenic and suspected to be toxic to reproduction.
3. Fillers (up to 50) *	Calcium carbonate	[44]	207-439-9	** According to the ECHA in REACH registrations, this substance causes serious eye damage, causes skin irritation and may cause respiratory irritation.
4. Reinforcements (15–30) *	Carbon fibers	[44]	231-153-3	According to the ECHA in CLP notifications, this substance causes serious eye irritation, is self-heating in large quantities and may catch fire and may cause respiratory irritation.

ECHA—European Chemical Agency; CLP—classification, labeling, and packaging. * Typical amount range (% *w*/*w*) [44]. ** Substances classified as dangerous and substances of very high concern (SVHC). Source: authors.

Plastics have the potential to leach the chemicals that comprise them, potentially causing serious harm to human health. The most well known of these chemicals is bisphenol A (BPA), which was first used as a synthetic estrogen in the 1930s. Other chemicals in plastic, such as phthalates, are often used as softeners for polyvinyl chloride (PVC) plastic to make plastic more flexible. But phthalates are harmful to human health. Bis(2-Ethylhexyl) phthalate (DEHP), benzyl butyl phthalate (BBP), dibutyl phthalate (DBP), and diisobutyl phthalate (DIBP) are classified as endocrine disruptors that are toxic to reproduction, which means that they may damage fertility and impact fetal development [45]. Both BPAs and phthalates are found in plastic containers used for food distribution and consumption available in the market today. Styrene, which is found in single-use polystyrene products, is a neurotoxin that has been found to leach into the foods and beverages being held in the polystyrene container.

The risks related to the consumption of food held in single-use plastic containers have been arguably emerging given the limited assessments pre-consumer deployment. These considerations are likely aligned to speed to market and pro-growth market perspectives that dominate country economic policies [46]. However, the increased attention to sustainable consumption has promoted scrutiny of the life cycle of single-use plastics; for example, the toxic nature of plastic leachate is increasingly being studied as a public health threat [47].

The regulatory challenges associated with food-contact plastics, particularly concerning safety, stem from the complex interplay between material innovation, risk assessment, and evolving policy frameworks. Conventional plastics have long been scrutinized for potential chemical migration—such as endocrine-disrupting phthalates or bisphenols—into food, prompting stringent but often fragmented global standards. The rise of bio-based and biodegradable plastics introduces additional complications, as their novel formulations may lack sufficient toxicological data or standardized testing protocols to ensure long-term safety. Regulatory bodies struggle to keep pace with these advancements, leading to inconsistencies in approval processes, labeling requirements, and permissible thresholds for migrating substances. Furthermore, the absence of harmonized international regulations creates trade barriers and consumer confusion, while composting and recycling infrastructures often fail to distinguish between food-safe and non-food-safe bioplastics, risking contamination. Existing policy frameworks for bioplastics are inadequate because they fail to address critical gaps in material standardization, end-of-life infrastructure, and consistent safety assessments, leading to regulatory fragmentation and unintended environmental trade-offs. Addressing these challenges requires not only updated safety assessments for emerging materials but also enhanced collaboration between scientists, policymakers, and industry to align innovation with public health priorities without stifling sustainability progress.

Considering the above, this article aims to respond to the following research questions:

**RQ1.** How do current EU policy instruments address the specific risks and sustainability requirements of bio-based and biodegradable plastics in food packaging?

**RQ2.** What are the gaps between the environmental claims and real-world end-of-life outcomes of bio-based food packaging materials under existing waste management infrastructures?

**RQ3.** To what extent do current bio-based plastics used in food packaging comply with food safety and toxicity standards under European regulations?

## 2. Identifying Bio-Based or/and Biodegradable Plastics as Sustainable Alternatives

Because plastic pollution poses a significant problem for the environment and human health, plastic waste has become a focus both for science and policy. Some approaches to handle plastic pollution are related to the consumption stage (e.g., bans, taxes), while others address the plastic with respect to waste management (e.g., by improving collection, controlling landfills) [48]. The use of alternatives, primarily bioplastics [1,49], has also garnered sizable interest as a substitute. Significant plastic waste has become a focus both for science and policy bans, while others address plastic with respect to its primary use and have also garnered sizable interest as a substitute.

Due to the complexity of the production and use of bioplastics—including the aspects of biodegradable and bio-based plastics—clear policies and scientific findings regarding their implementation are still under development. It is significant to note that bioplastics are bio-based polymers that are produced from renewable resources, including carbohydrates, vegetable oils, etc., in the presence of microorganisms [50]. Marketed as an alternative to plastic source they do have similar physical properties which may promote rapid substitution and overuse, parallelling the deployment and consumption history of the petroleum-based plastic they are targeted to replace. Specifically, not all bioplastics are the same, and varying chemical compositions can pose an issue at disposal, even composting [51]. As a result, it remains too early to determine final conclusions regarding the environmental impact of bio-based and biodegradable plastics [52].

Bishop et al. [49] highlight the fact that several studies of life cycle impacts of bioplastics do not constitute a full assessment, potentially creating misleading information and increasing controversy on the topic. Although some studies suggest that bio-based plastics might have an even higher negative effect on the environment [53], several other studies have found that the environmental impact is lower with respect to climate change [54]. Since bio-based plastics, under the appropriate conditions, can degrade in a shorter period of time, the most suitable method for disposal is composting; their use eliminates the food contamination issue for plastic recycling [55] with respect to, under the appropriate conditions, their use eliminating the food contamination issue for plastic recycling.

**Figure 1:**
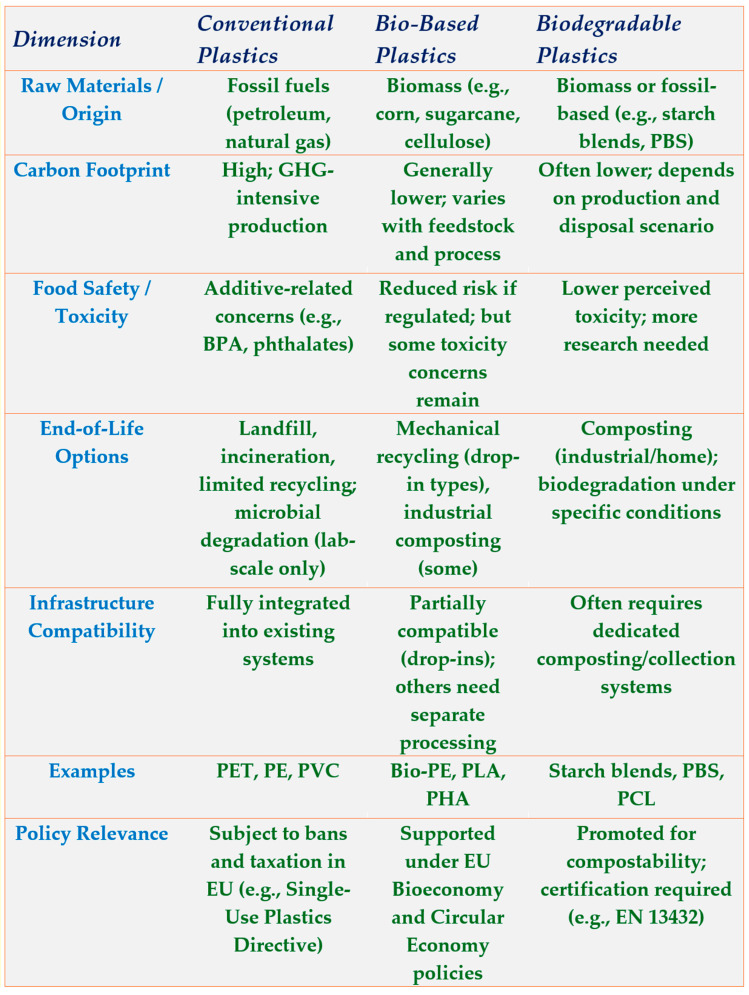
Comparative overview of plastic categories relevant to food packaging, including conventional, bio-based, and biodegradable plastics. Key sustainability and policy dimensions are presented: origin, carbon footprint, food safety, end-of-life options, infrastructure compatibility, material examples, and regulatory context. BPA—bisphenol A; EN 13432—European Standard 13432 [59]; EU—European Union; GHG—greenhouse gas; PCL—polycaprolactone; PE—polyethylene; PET—Polyethylene terephthalate; PHA—polyhydroxyalkanoates; PLA—polylactic acid; PBS—polybutylene succinate; PVC—polyvinyl chloride. Source: authors.

Further contributing to the emerging understanding of bioplastic impacts, researchers have noted per- and polyfluoroalkyl (PFAS) contamination resulting from bioplastics [56], as well as general toxicity of the product [57]. PFAS poses significant health and environmental risks due to their persistence, bioaccumulation, and potential toxicity, necessitating mitigation strategies such as stricter regulatory bans, development of safer alternative materials, enhanced wastewater treatment technologies, and improved product labeling to minimize exposure [58]. Specifically, alternatives to plastic must address the potential externalities of composting, incineration, and reuse. Figure 1 presents a comparative matrix of conventional, bio-based, and biodegradable plastics, focusing on aspects relevant to food packaging such as carbon footprint, toxicity, end-of-life options, and compatibility with existing infrastructure. It allows a comparable overview to inform policy and material selection decisions.

## 3. The Role of the Policies in Fostering Bio-Based Alternatives to Plastic

Policies play a crucial role in fostering the development and adoption of bio-based alternatives to plastic. Plastic pollution has become a significant environmental concern, and transitioning to bio-based alternatives can help mitigate its negative impacts. The European Union (EU) has been at the forefront of environmental policy, particularly in fostering the development and adoption of bio-based alternatives to conventional plastics. These efforts are part of a broader strategy to reduce plastic waste, mitigate climate change, and transition towards a circular economy. European policies play a critical role in this context by creating regulatory frameworks, setting targets, providing financial incentives, and supporting research and innovation.

**Figure 2:**
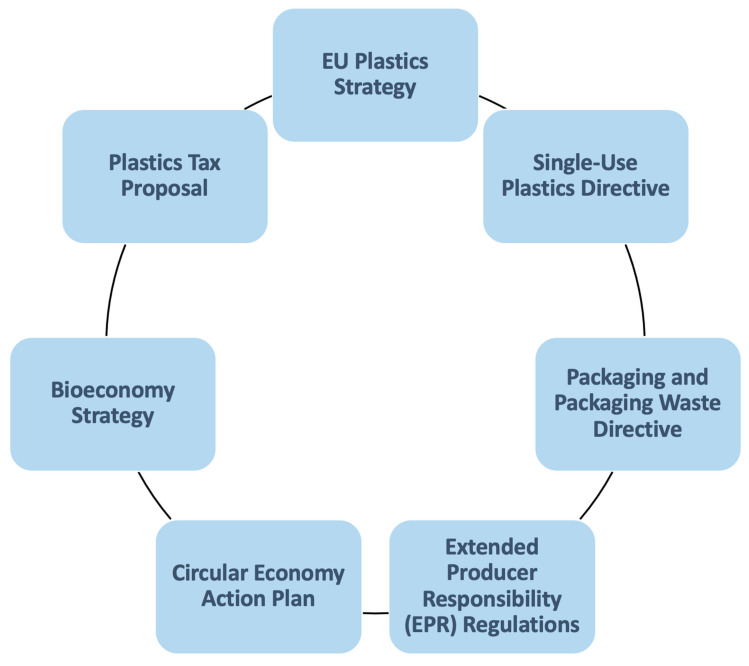
Overview of key EU policies addressing plastic reduction and bio-based alternatives. The figure highlights legislative instruments most relevant to food packaging contexts, indicating varied alignment with safety, circularity, and implementation feasibility. Source: own authors.

Figure 2 synthesizes the major EU policy instruments shaping the shift from conventional to sustainable plastic use, especially in food packaging. Rather than listing aims alone, each policy’s relevance to core sustainability criteria, notably food safety, circularity, and industry uptake, is assessed. The EU Plastics Strategy is a framework guiding the EU’s efforts to address the challenges posed by plastic waste, a strategy outlining ambitious targets for recycling, reduction, and the transition to a circular economy. The Single-Use Plastics Directive is a targeted policy addressing the environmental impact of single-use plastic products, aiming to reduce the consumption of specific plastic items, such as cutlery, straws, and plates, by promoting more sustainable alternatives. The Packaging and Packaging Waste Directive focuses on minimizing the environmental impact of packaging, establishing guidelines for the design and use of packaging materials. It encourages the adoption of bio-based and recyclable materials, contributing to a more sustainable packaging industry. The Extended Producer Responsibility (EPR) Regulations place the onus on producers to manage the entire life cycle of their products, including collection, recycling, and proper disposal. EPR encourages producers to adopt eco-friendly materials and designs. The Circular Economy Action Plan is a broader initiative promoting the transition from a linear “take-make-dispose” model to a circular economy. The plan emphasizes sustainable product design, recycling infrastructure improvement, and fostering the market for recycled materials. The Bioeconomy Strategy encourages the development and use of bio-based alternatives to conventional plastics, aligning with the EU’s commitment to a more sustainable and resource-efficient future. Finally, the Plastics Tax Proposal is an upcoming policy aimed at incentivizing the use of recycled plastics and discouraging the consumption of virgin plastics through a taxation mechanism.

These policies differ in scope and impact. The Single-Use Plastics Directive directly targets food-related items such as cutlery and containers, reducing environmental and human exposure to plastic residues. The Packaging and Packaging Waste Directive promotes eco-design but offers limited clarity on bio-based vs. conventional materials. The Bioeconomy Strategy supports innovation, but its link to food safety remains indirect. Only Extended Producer Responsibility (EPR) mechanisms actively address life cycle responsibility, yet food packaging-specific obligations are rare. Thus, alignment with food safety and food system resilience remains partial across most instruments.

The main forms of packaging waste management include landfilling, recycling, incineration with energy recovery, composting, and reuse systems. Landfilling remains the most common but least sustainable method, as it contributes to long-term environmental pollution and resource wastage. Recycling processes materials like plastics, metals, and paper into new products, though contamination and inconsistent collection systems often limit its effectiveness. Incineration reduces waste volume while generating energy, but concerns persist over air emissions and toxic ash residues. Composting is suitable for biodegradable and bio-based packaging, provided proper industrial facilities exist to prevent contamination with conventional plastics. Reuse systems, such as refillable containers and deposit schemes, offer a circular alternative by extending product lifecycles, though they require consumer participation and logistical infrastructure. Each method presents trade-offs in environmental impact, economic feasibility, and scalability, highlighting the need for integrated waste management policies tailored to regional capabilities and material-specific challenges.

The policies in Figure 2 collectively reflect the EU’s commitment to combating plastic pollution and steering industries and consumers toward environmentally friendly practices. Figure 2 serves as a visual guide to the diverse range of initiatives aimed at fostering a more sustainable and circular approach to plastic use in the European Union.

**Figure 3:**
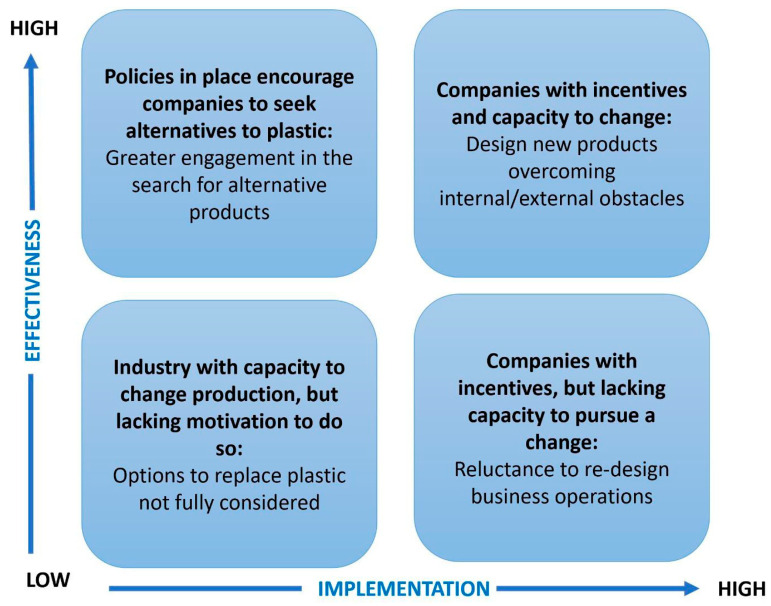
Potential impacts of plastic-related policies on sustainability outcomes, particularly in food packaging. Source: own authors.

As far as the growth of bio-based alternatives to plastic is concerned, some of the ways in which policies can support progress in this field are detailed below (see Figure 3):

Mobilizing research and development funding. Governments can allocate funds to support research and development in the field of bio-based materials to ensure that products are safe for humans and the environment across their life cycle. This funding can be used to explore and improve the production processes, scalability, and performance of bio-based alternatives to plastic. It can also help in developing new technologies and materials that are sustainable, cost-effective, and have a reduced environmental footprint.

Mobilizing incentives and subsidies. Governments can provide financial incentives and subsidies to encourage the adoption of bio-based alternatives. These incentives can include tax breaks, grants, or subsidies for businesses that invest in the development, production, or use of bio-based materials. Such measures can help reduce the cost barriers associated with transitioning from traditional plastics to bio-based alternatives, making them more economically viable.

Setting up regulations and standards. Policymakers can establish regulations and standards that promote the use of bio-based alternatives to plastic. Standards should require life cycle assessments to evaluate holistic impacts related to bio-based products. Further, regulations could include setting targets for the percentage of bio-based content in products, implementing labeling requirements to inform and educate consumers on the benefits of transitioning away from traditional plastics, or imposing restrictions on the use of certain types of plastics. Clear and consistent regulations provide certainty to businesses and create a level playing field for the bio-based industry.

Encouraging specific procurement policies. Governments can lead by example by incorporating bio-based alternatives into their own procurement policies. By mandating or giving preference to bio-based materials in public procurement, governments can create a significant market demand that encourages investment and innovation in the bio-based sector. This, in turn, can drive economies of scale, reduce costs, and improve the availability of bio-based alternatives in the market.

Figure 3 provides an overview of some of the potential impacts of current and future policies to handle plastics.

Additionally, policymakers can foster collaboration between industry, academia, and research institutions to facilitate knowledge exchange and innovation in the bio-based materials sector. This can include establishing public-private partnerships, supporting technology transfer, and creating platforms for stakeholders to share best practices and experiences. Collaborative efforts can accelerate the development and commercialization of bio-based alternatives. Policies should also prioritize public awareness and education campaigns to inform consumers about the benefits of bio-based alternatives and how to properly dispose of them. Public awareness is thus crucial in elucidating plastic pollution and related environmental consequences in order to encourage citizens towards eco-friendly products and more sustainable food packaging choices [4]. By promoting responsible consumption and waste management practices, policymakers can encourage individuals to choose bio-based alternatives and contribute to reducing plastic pollution. Overall, policies that provide financial support, create market demand, establish regulations, and promote collaboration are essential for fostering the development and adoption of bio-based alternatives to plastic. By addressing the environmental challenges associated with traditional plastics, these policies can contribute to a more sustainable and circular economy.

Apart from outlining the benefits from the potentials of bio-based plastics production, there is a need for “niche policies” based on the banning of some products (e.g., single-use carrier plastic bags or plastic cutlery, as the European Union has implemented from July 2021) to be combined with education and a wide promotion of reusable materials. There is also a need for a smart set of “action-oriented” policies, which may further advance scientific progress and trigger more investments from the private sector. This is essential to allow for sustainability-focused bioplastics, which may be reused many times and recycled when the end of their life has been reached, limiting their life cycle impact. According to Keranen [60], bioplastics can be seen as a potential innovation in advancing sustainable development in food packaging [60]. Bioplastics can be seen as a potential innovation in advancing sustainable development in food packaging. New bio-based and biodegradable plastics offer opportunities to better preserve food, extend shelf life, and enable improved separation and recycling of food waste. Agricultural products and food by-products such as corn, cassava, and rice straw [5] are increasingly used as raw materials for bioplastics [1], reinforcing the circular (bio)economy through valorization of food system wastes [61]. The environmental problems caused by plastic waste, together with the huge cost of bioplastics being developed, also stimulate the research on green composites aiming to be used as green fillers (replacing up to 20% of bioplastic) and valorizing agriculture and marine waste, reducing bioplastics’ cost and improving aesthetics, without entirely compromising their mechanical properties, generally inferior [9,62], and environmental performance, in compliance with the circular economy [63].

Indeed, an intelligent mix of policies may further research and development (R&D), trigger the development of innovative technologies, and, inter alia, increase the demand for bio-based materials. These are strategic times in the sense that the European Commission is currently revising and considering new policies in the field of bio-based products. Appropriate policy support now may pave the way for progress in the next 20 years, accelerating the transitions from a fossil-fuel-based economy to a bio-based one foundationally incorporating a “do no harm” approach.

Bioeconomy must consider the sustainability of production and consumption systems by replacing fossil fuels with biomass across industries. In specific regions of the world such as Brazil, China, Europe, the United States (US), and Thailand, considered to be five major crop-based bioplastics producers, the solution to incentivizing bioplastics production targets would pass by stimulating production subsidies, following the examples of biofuel mandates, aiming to attend sustainability metrics such as land footprint or greenhouse gas (GHG) savings from fossil fuel substitution, as examples, necessarily moving to more advanced technologies, value chains, and business models to contribute to Sustainable Development Goals (SDGs) [64]. This means that efforts may be undertaken to pursue a bio-based economy, including bioplastic materials, without encouraging excessive consumption and its externalized impacts (i.e., littering) and without contributing further to pollution, which already is a serious global problem. Fundamental to the needed changes is that production and consumption be aligned to Earth systems and consider the need for biodiversity, thereby limiting the human impact on the planet from the product development stage onward. What should not be omitted from the discussion is a focus on eliminating toxicity to the air, land, and groundwater in the pursuit of environmentally friendly alternatives to plastics.

## 4. Conclusions

The increasing production and use of bio-based and biodegradable plastics present both a challenge and an opportunity, particularly within the context of food packaging, where human exposure and environmental risks intersect. As this article has outlined, plastic pollution is not only a waste management issue but also a direct threat to ecosystems, food chains, and public health. The persistence of petroleum-based plastics, their chemical additives, and their inadequate disposal methods—often intersecting with food packaging applications—demands systemic change. Bio-based alternatives have emerged as a promising route toward reducing dependence on fossil-derived materials and mitigating pollution.

Yet, their benefits cannot be assumed. Bio-based plastics still face concerns regarding toxicity, compostability under real-world conditions, environmental trade-offs, and lifecycle impacts. In food systems, these concerns are amplified: packaging must not only preserve safety and shelf life but must also avoid chemical leaching or contamination. As such, food-contact bio-based materials require specific attention in the policy landscape.

Aiming to address the specific challenges of food-contact bioplastics, the following policy recommendations are proposed:

▪ Adopt harmonized EU food-contact safety standards for bio-based materials, including specific thresholds for chemical migration, toxicity testing, and labeling.
▪ Mandate lifecycle toxicity and environmental risk assessments for all food-contact bioplastics, ensuring regulatory approval reflects real-world end-of-life conditions such as composting or incineration.
▪ Expand EPR to include design-for-safety principles for food-contact applications, prioritizing reuse and non-toxic materials.
▪ Create certification and labeling schemes (e.g., “Safe for Food Use—Biobased”) to guide industry innovation and consumer decision-making.
▪ Support public procurement policies that favor certified food-safe bioplastics, stimulating market demand for compliant, sustainable alternatives.

These measures aim to fill existing regulatory gaps while promoting innovation that aligns with human health, environmental integrity, and circular economy goals in food packaging.

Although it provides a comprehensive overview of the current situation and relevant recommendations, this article has some limitations. First, it primarily focuses on policy frameworks in developed nations, potentially overlooking challenges in developing economies where infrastructure and enforcement differ. Second, the analysis relies on the existing literature and policy documents, which may not fully capture real-world implementation barriers. Third, the paper does not extensively address economic trade-offs, such as the cost competitiveness of bio-based plastics versus conventional plastics. Lastly, stakeholder perspectives (e.g., industry, consumers) are underrepresented, limiting insights into practical adoption challenges.

Despite these limitations, the paper provides a welcome addition to the literature by highlighting the misalignment between bio-based plastics policies and circular economy principles. It underscores the need for integrated policy approaches that consider lifecycle impacts, rather than isolated material substitution. For practice, the findings emphasize the importance of harmonizing regulations across regions to avoid market fragmentation. Policymakers can use this research to design incentives for bio-based plastics while ensuring waste management systems adapt to new materials. Additionally, businesses in the food packaging sector may benefit from clearer regulatory guidance to align innovation with sustainability goals.

Future research should explore how bio-based plastics policies function in developing economies with different waste management infrastructures. Also, public perception and willingness to pay for bio-based food packaging should be further investigated, along with cost analyses and scalability challenges of bio-based alternatives.

Overall, to accelerate the transition toward sustainable packaging, governments should implement a set of policies. For instance, a tax incentive and certification program to encourage businesses to adopt bioplastics. Also, establish a national bioplastics certification system (e.g., “Certified Bio-Based” or “Industrially Compostable”) to ensure transparency, prevent greenwashing, and guide consumer choices. Furthermore, policies should support R&D by allocating funding to support research into next-generation bioplastics with improved durability, scalability, and cost-effectiveness, particularly for food and medical packaging.

Food packaging represents a high-leverage point for addressing the plastic crisis while advancing circular bioeconomy goals. By aligning regulatory action, innovation, and sustainable materials development, Europe and other regions can lead a transition that not only reduces plastic pollution but also enhances food safety, public health, and environmental integrity. Therefore, this article offers a timely contribution by integrating toxicity, regulatory gaps, and sustainability in the context of food-contact bioplastics. As material innovation continues to outpace policy, future efforts must focus on bridging this gap through interdisciplinary research and coordinated policy action that safeguards both human health and environmental outcomes.

## Data Availability

No new data were created or analyzed in this study. Data sharing is not applicable to this article.
